# Supplementary material for: Hydrophobicity of arginine leads to reentrant liquid-liquid phase separation behaviors of arginine-rich proteins
Source: Nat Commun. 2022 Nov 28;13:7326. doi: 10.1038/s41467-022-35001-1 (PMC9705477; doi:10.1038/s41467-022-35001-1)
Supplement: Supplementary file 1 — Supplementary information [file 41467_2022_35001_MOESM1_ESM.pdf]

Supplementary Information

Hydrophobicity of arginine leads to reentrant liquid-liquid phase separation behaviors of arginine-rich proteins

Yuri Hong[1], Saeed Najafi[2], Thomas Casey[2], Joan-Emma Shea[2]*, Song-I Han[2,3]*, Dong Soo Hwang[1,4]*

[1] School of Interdisciplinary Bioscience and Bioengineering, Pohang University of Science and Technology (POSTECH), Pohang 37673, Republic of Korea
[2] Department of Chemistry & Biochemistry, University of California, Santa Barbara, California 93106, United States
[3] Department of Chemical Engineering, University of California, Santa Barbara, California 93106, United States
[4] Division of Environmental Science and Engineering, Pohang University of Science and Technology (POSTECH), Pohang 37673, Republic of Korea

The Supplementary Information includes:

Supplementary Information for Hofmeister effect in the simple coacervation of protamine (Supplementary Figure 1)
Supplementary Information for UCST behavior of low salt HA-Protamine coacervation (Supplementary Figure 2)
Supplementary Information for indirect umbrella sampling (INDUS) simulations (Supplementary Figure 3)
Supplementary Information for coalescence events of HA-Protamine coacervates (Supplementary Figure 4)
Supplementary Information for spin labeled state used in EPR and ODNP (Supplementary Figure 5)
Supplementary Information for all EPR spectra (Supplementary Figure 6)
Supplementary Information for atomistic molecular dynamics simulations (Supplementary Figure 7)
Supplementary Information for conformational properties of pseudo-protamine-RtoK and poly-arginine (Supplementary Figure 8)
Supplementary Information for RGG/RG motif-containing SG proteins (Supplementary Figure 9)
Supplementary Information for turbidity of HA-Protamine and HA-εPL at different mixing ratios (Supplementary Figure 10)
Supplementary Information for water fluctuation over time (Supplementary Figure 11)

Supplementary information for Hofmeister effect in the simple coacervation of protamine (Supplementary Figure 1)

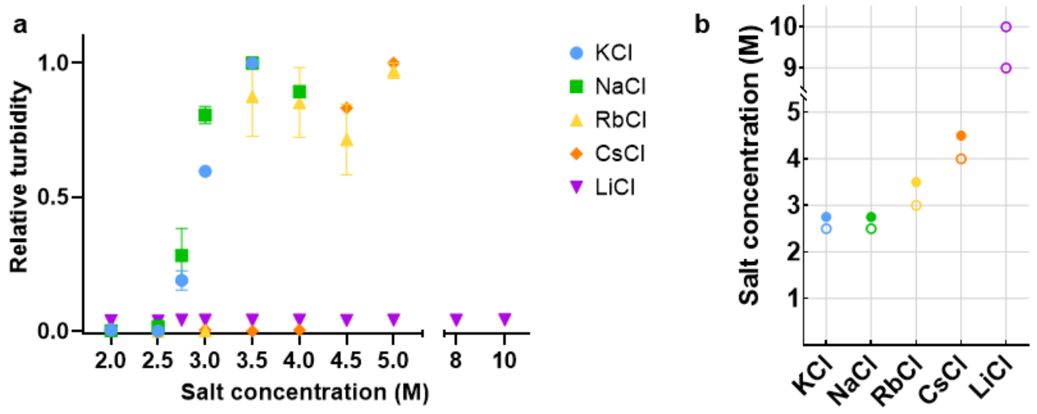

Supplementary Figure 1. Effect of Hofmeister series on simple coacervation of protamine. a Turbidity of protamine simple coacervation at specific salt concentrations. **b** Open circle indicates no phase separation. The closed circle indicates phase separation. Protamine concentration is 10 mg/mL. Measurement was performed in triplicate. N = 3 independent experiments except for LiCl data (n = 1). Data are presented as mean values +/- SD. The data are available in the Figshare repository under [https://doi.org/10.6084/m9.figshare.21509343.v3].

Supplementary Information for UCST behavior of low salt HA-Protamine coacervation (Supplementary Figure 2)

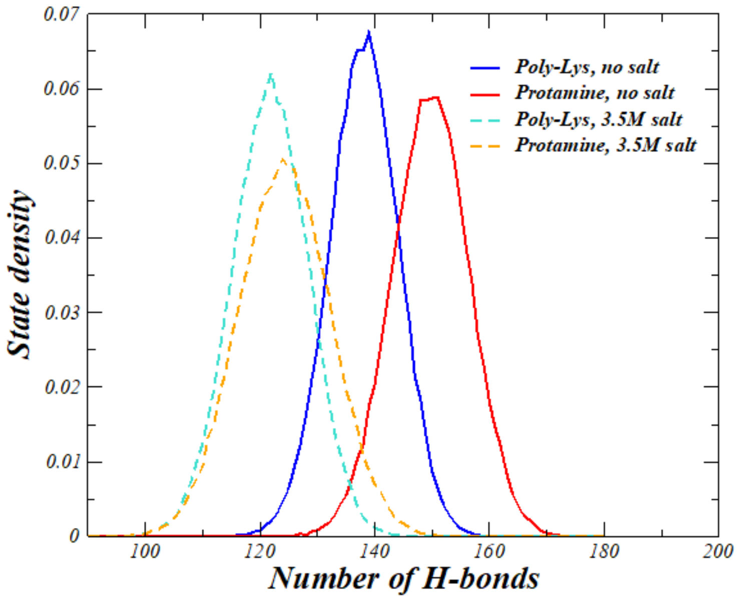

Supplementary Figure 2. The probability distribution of the total hydrogen bonds between the hydration water and poly-Lysine, and the total hydrogen bonds between the hydration water and protamine without/with salt.

Supplementary Information for indirect umbrella sampling (INDUS) simulations (Supplementary Figure 3)

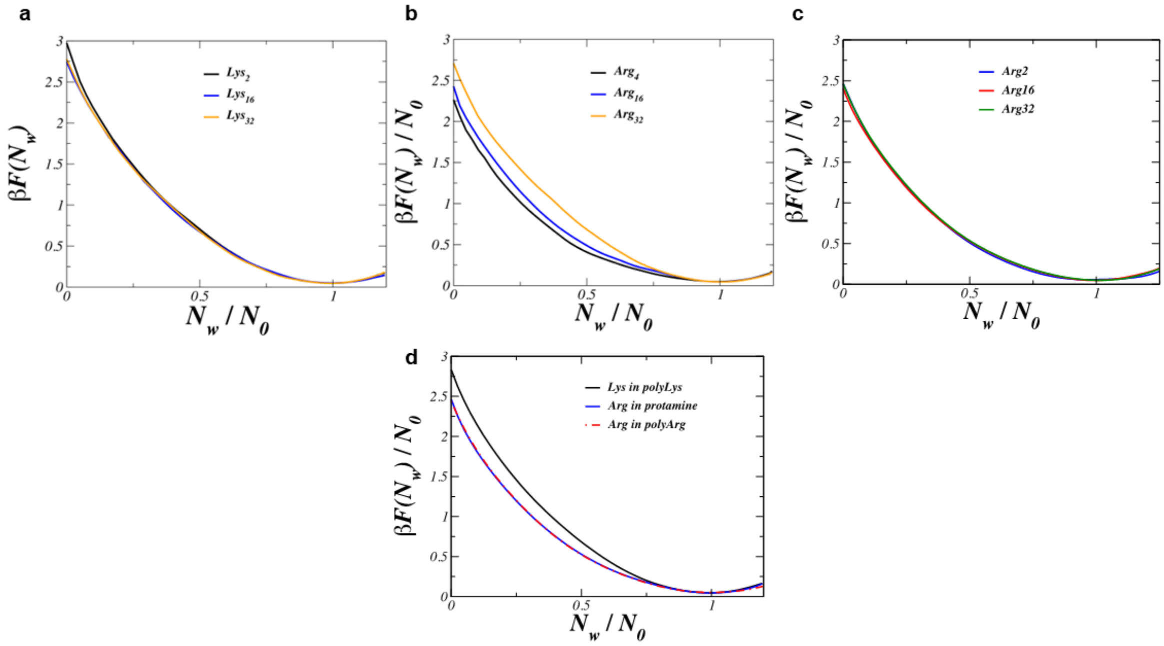

Supplementary Figure 3. The dehydration free-energy obtained from INDUS. a Dewetting free energies per water molecule that are independently calculated in the hydration volume of Lys_2, Lys_{16} and Lys_{33}. **b**, The same quantity is characterized in the hydration volume of Arg_4, Arg_{16} and Arg_{33} of protamine chain. **c** The same quantity is characterized in the hydration volume of Arg_4, Arg_{16} and Arg_{33} of poly-Arg chain. **d** Average dewetting free energies in the polyLys, protamine, and poly-Arg. Panel a, the Lys hydrophobicity in the homopolymer poly-Lys does not change across different sites and locations along the peptide. The calculated free energies feature negligible errors, i.e., less than 0.03kT (characterized by bootstrapping). In fact, we observe similar behavior if we investigate the Arg residue hydrophobicity in the homopolymer polyArg (panel c). In contrast, we see that the Arg dewetting free energy is a context dependent parameter in the protamine chain that is composed of different types of residues. This indicates that depending on the type of the adjacent residues, the hydrophobicity of Arg can change. We note however that the average dewetting free energies (over the three investigated residues: 4, 16, 32) in the poly-Arg and protamine peptides are still identical (panel d).

Supplementary Information for coalescence events of HA-Protamine coacervates (Supplementary Figure 4)

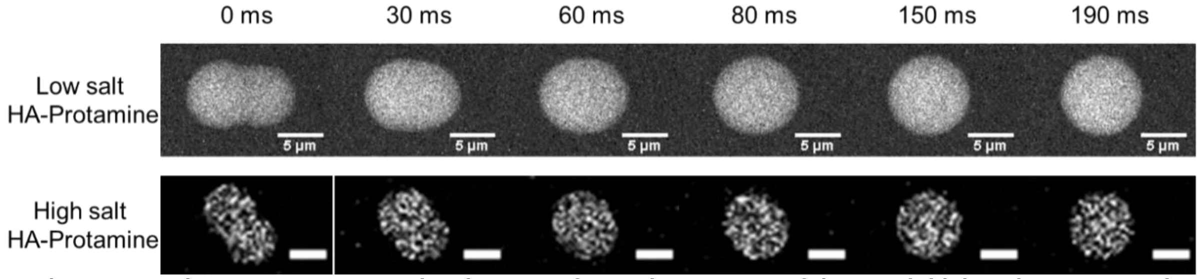

Supplementary Figure 4. Representative images of merging events of low and high salt HA-Protamine coacervates in time series. Scale bar = 5 µm. Merging events were observed 9 and 7 times for low and high salt HA-Protamine, respectively.

Supplementary Information for spin-labeled state used in EPR and ODNP (Supplementary Figure 5)

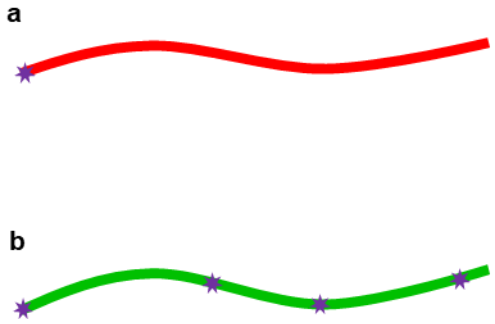

Supplementary Figure 5. Illustrations of spin-labeled proteins. a Protamine is spin-labeled only on the N-terminal amine. **b** ε-Poly-L-lysine is randomly spin-labeled on multiple amines.

Supplementary Information for all EPR spectra (Supplementary Figure 6)

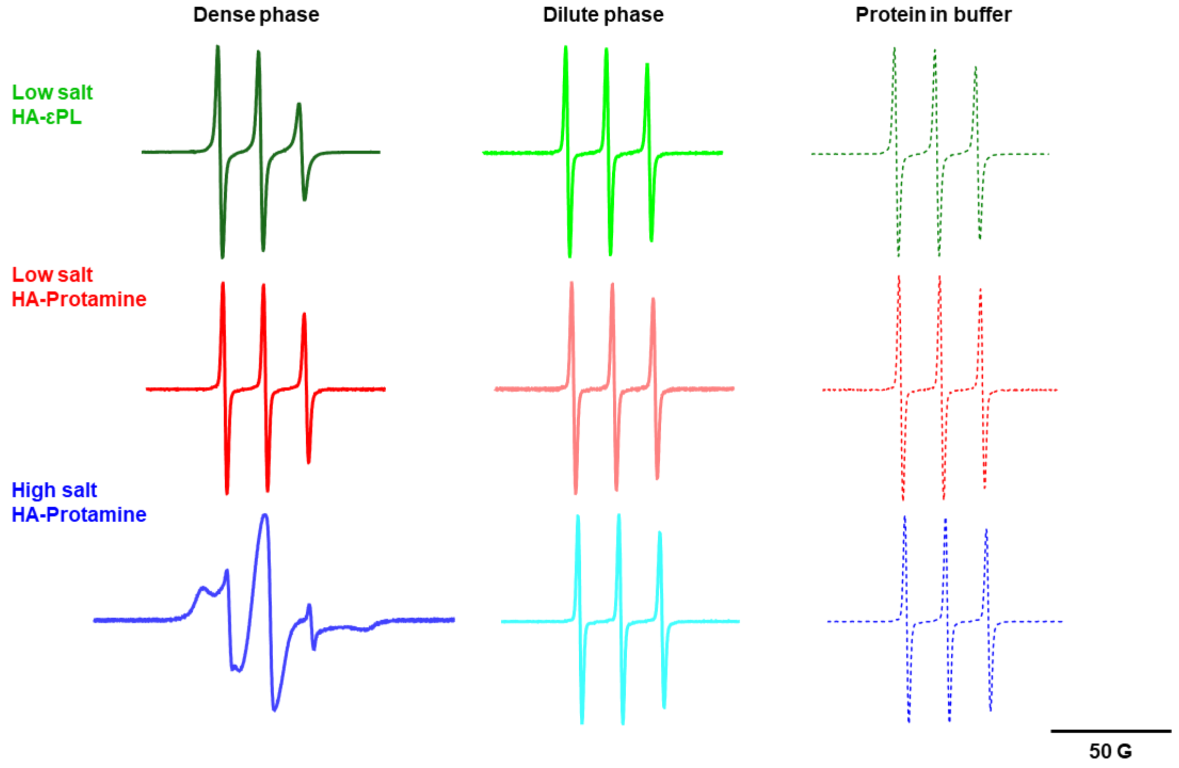

Supplementary Figure 6. EPR spectra of SL-εPL and SL-protamine in dense phase, dilute phase and low/high buffer (without coacervation). The data are available in the Figshare repository under [https://doi.org/10.6084/m9.figshare.21509343.v3].

Supplementary Information for atomistic molecular dynamics simulations (Supplementary Figure 7)

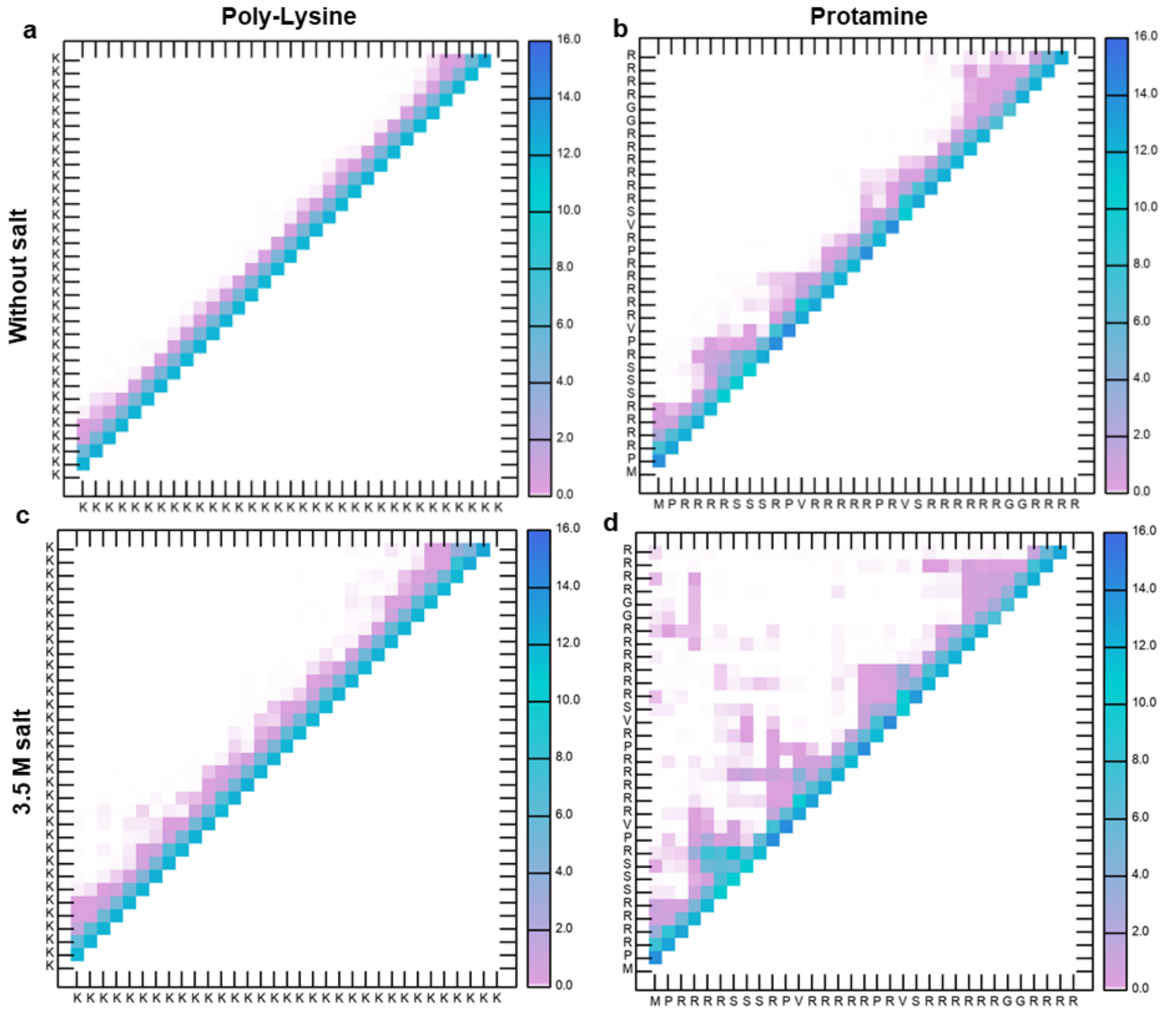

Supplementary Figure 7. Contact map distribution. a and **b** show the intramolecular contact map of poly-lysine and protamine chains without salt. **c** and **d** describe the intramolecular contact map of poly-lysine and protamine chains in presence of 3.5 M salt in solution. The data are available in the Figshare repository under [https://doi.org/10.6084/m9.figshare.21509343.v3].

Supplementary information for conformational properties of pseudo-protamine-RtoK and poly-arginine (Supplementary Figure 8)

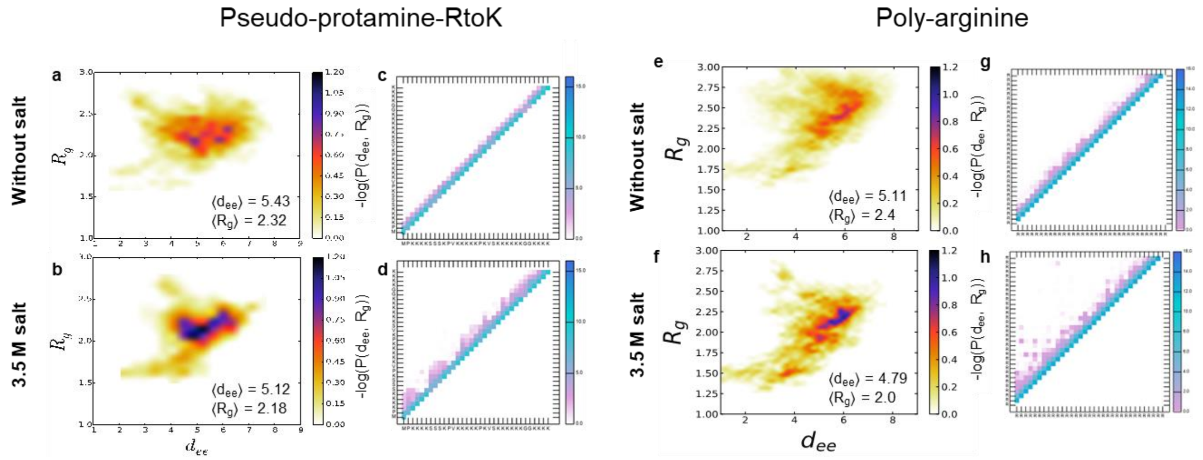

Supplementary Figure 8. Conformational properties of pseudo-protamine-RtoK and poly-arginine. a, e and **b, f** represent the probability distribution of the radius of gyration (R_g) and the end-to-end (d_{ee}) distance without and with salt, respectively. **c**, **g** and **d, h** show the intramolecular contact map without salt and in the presence of salt, respectively. The data are available in the Figshare repository under [https://doi.org/10.6084/m9.figshare.21509343.v3].

Supplementary Information for RGG/RG motif-containing SG proteins (Supplementary Figure 9)

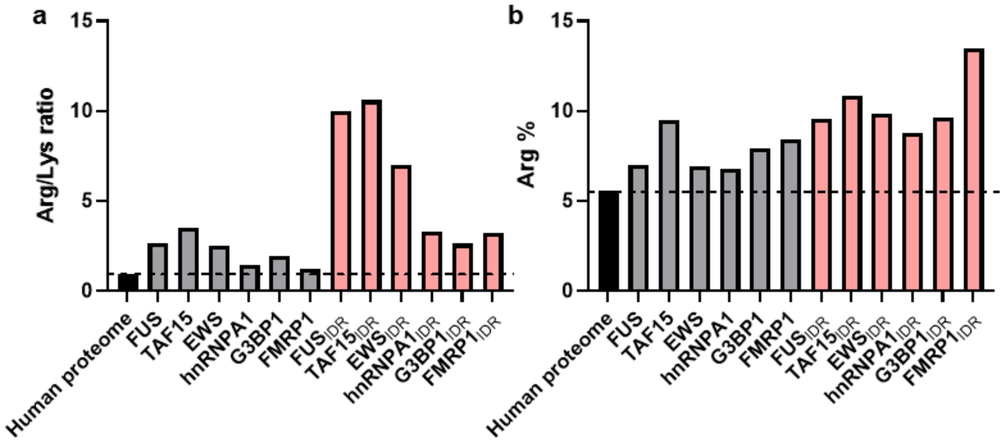

Supplementary Figure 9. RGG/RG motif-containing proteins in stress granules. a Arginine to lysine ratio of the full sequence (grey) and intrinsically disordered regions (IDRs) (pink) of the RGG/RG motif-containing SG proteins compared to the arginine to lysine ratio of the human proteome. **b** Arginine percentage of the full sequence (grey) and IDRs (pink) of the RGG/RG motif-containing SG proteins compared to the arginine percentage of the human proteome. Human proteome database is obtained in UniProt Knowledgebase (UniProtKB)[1] (Proteome ID UP000005640 [https://www.uniprot.org/proteomes/UP000005640]). IDRs is determined based on the UniProtKB database[1]. IDRs (amino acid sequence) of FUS (RNA-binding protein FUS, P35637 [https://www.uniprot.org/uniprotkb/P35637/entry]): 1-286/375-424/444-526, TAF15 (TATA-binding protein-associated factor 2N, Q92804 [https://www.uniprot.org/uniprotkb/Q92804/entry]): 1-237/324-356/373-592, EWS (Ewing sarcoma breakpoint region 1 protein, Q01844 [https://www.uniprot.org/uniprotkb/Q01844/entry]): 123-360/448-525/547-656, hnRNPA1 (Heterogeneous nuclear ribonucleoprotein A1, P09651 [https://www.uniprot.org/uniprotkb/P09651/entry]): 182-240/317-372, G3BP1 (Ras GTPase-activating protein-binding protein 1, Q13283 [https://www.uniprot.org/uniprotkb/Q13283/entry]): 144-172/184-431/255-329/413-466, FMRP1 (Fragile X mental retardation protein 1, Q06787 [https://www.uniprot.org/uniprotkb/Q06787/entry]): 325-349/443-632. The data are available in the Figshare repository under [https://doi.org/10.6084/m9.figshare.21509343.v3].

Supplementary information for turbidity of HA-Protamine and HA-εPL at different mixing ratios (Supplementary Figure 10)

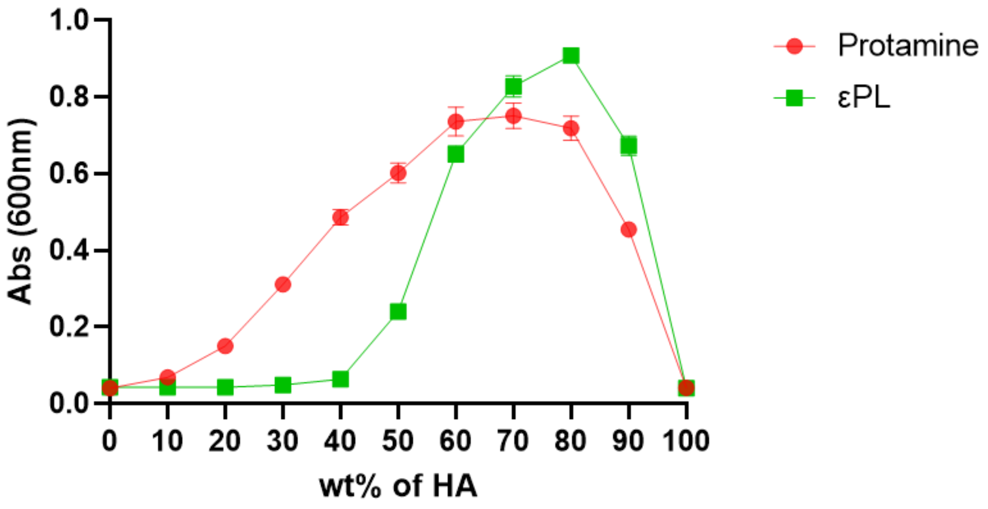

Supplementary Figure 10. Turbidity measurement of HA-Protamine and HA-εPL coacervation depending on the mixing ratio. Total concentration is 10 mg/mL. N=3 independent experiemtns, Data are presented as mean values +/- SD. The data are available in the Figshare repository under [https://doi.org/10.6084/m9.figshare.21509343.v3].

Supplementary Information for water fluctuation over time (Supplementary Figure 11)

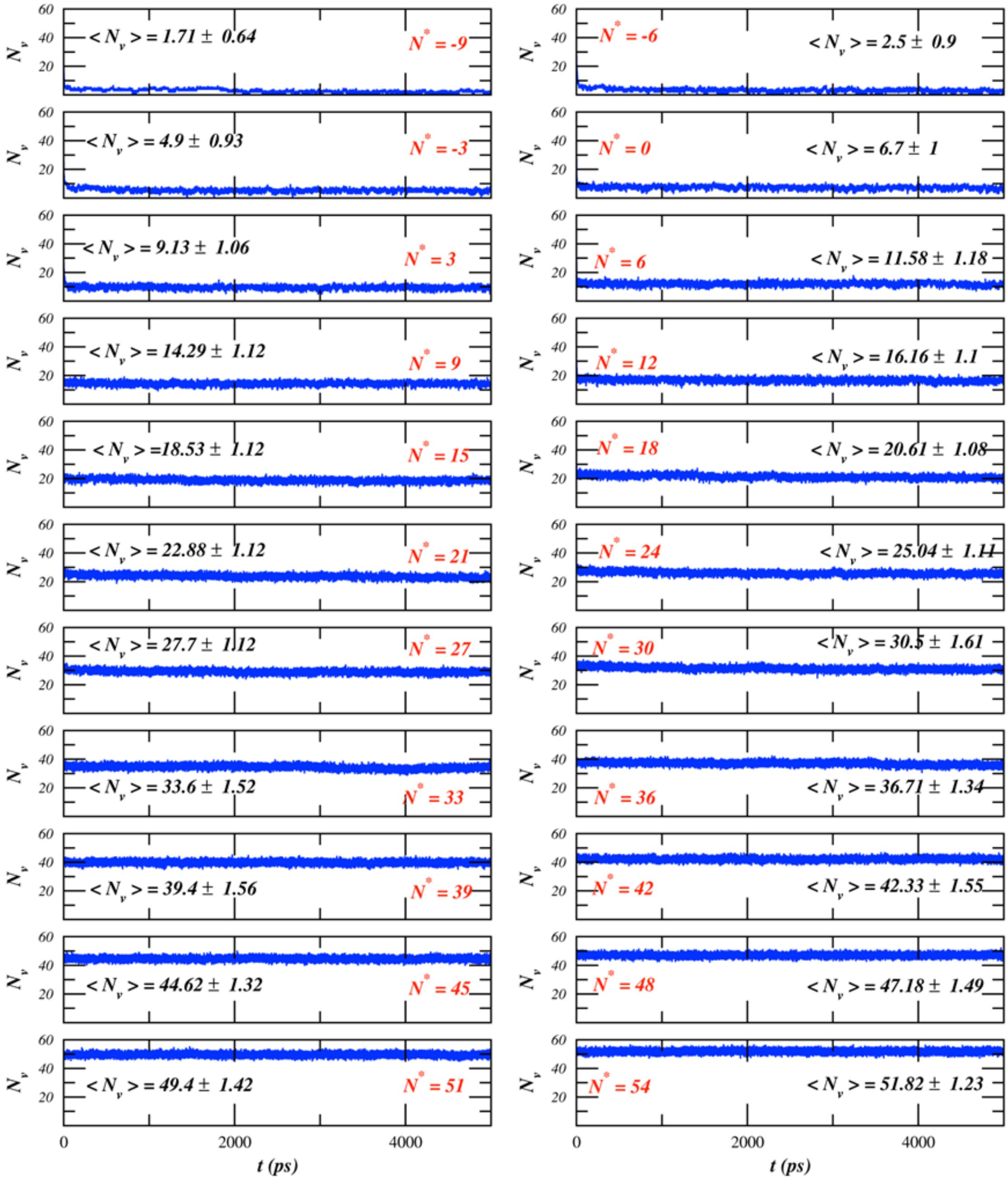

Supplementary Figure 11. The time series of the water fluctuation of the Arg16 of protamine at the different biased N* (the imposed total water molecules in the hydration volume). Note that Nv is the number of water molecules that we have after biasing N*. Considering that the average normalized water fluctuation (to N_0, the total hydration water at equilibrium; for Arg_{16} in protamine N_0 = 44), is ~0.03 across different biased N^*, the effective dewetting free energy fluctuation per-water molecule is only ~0.06 kT. It is to be noted that ~0.06 kT is 0.1 of the dewetting free energy difference between Arg and Lys.

Reference

[1] Bateman, A.. UniProt: the universal protein knowledgebase in 2021.*Nucleic Acids Research* **49,** D480–D489 (2021).
