# Supplementary material for: Hydrophobicity of arginine leads to reentrant liquid-liquid phase separation behaviors of arginine-rich proteins
Source: Nat Commun. 2022 Nov 28;13:7326. doi: 10.1038/s41467-022-35001-1 (PMC9705477; doi:10.1038/s41467-022-35001-1)
Supplement: Supplementary file 3 — Reporting Summary [file 41467_2022_35001_MOESM3_ESM.pdf]

nature portfolio

Corresponding author(s): Dong Soo Hwang

Last updated by author(s): Nov 9, 2022

Reporting Summary

Nature Portfolio wishes to improve the reproducibility of the work that we publish. This form provides structure for consistency and transparency in reporting. For further information on Nature Portfolio policies, see our Editorial Policies and the Editorial Policy Checklist.

Statistics

For all statistical analyses, confirm that the following items are present in the figure legend, table legend, main text, or Methods section.

n/a	Confirmed	
	x	The exact sample size (n) for each experimental group/condition, given as a discrete number and unit of measurement
	x	A statement on whether measurements were taken from distinct samples or whether the same sample was measured repeatedly
x		The statistical test(s) used AND whether they are one- or two-sided<br>*Only common tests should be described solely by name; describe more complex techniques in the Methods section.*
x		A description of all covariates tested
x		A description of any assumptions or corrections, such as tests of normality and adjustment for multiple comparisons
	x	A full description of the statistical parameters including central tendency (e.g. means) or other basic estimates (e.g. regression coefficient) AND variation (e.g. standard deviation) or associated estimates of uncertainty (e.g. confidence intervals)
x		For null hypothesis testing, the test statistic (e.g. F, t, r) with confidence intervals, effect sizes, degrees of freedom and P value noted<br>*Give P values as exact values whenever suitable.*
x		For Bayesian analysis, information on the choice of priors and Markov chain Monte Carlo settings
x		For hierarchical and complex designs, identification of the appropriate level for tests and full reporting of outcomes
x		Estimates of effect sizes (e.g. Cohen's d, Pearson's r), indicating how they were calculated

Our web collection on statistics for biologists contains articles on many of the points above.

Software and code

Policy information about availability of computer code

Data collection	All ODNP experimental data were processed using DNPLab, an open-source Phyton package (Timothy Keller, Thomas Casey, Yanxian Lin, Thorsten Maly, & jmfranck. (2021). DNPLab/DNPLab: (Version 2DODNPPaper). Zenodo. https://doi.org/10.5281/zenodo.4670553). In particular, data were processed and fit using the 'hydrationGUI' module. The INDUS code is open source and available on GitHub (https://github.com/patellab511/indus).
Data analysis	All ODNP experimental data were processed using DNPLab, an open-source Phyton package (Timothy Keller, Thomas Casey, Yanxian Lin, Thorsten Maly, & jmfranck. (2021). DNPLab/DNPLab: (Version 2DODNPPaper). Zenodo. https://doi.org/10.5281/zenodo.4670553). In particular, data were processed and fit using the 'hydrationGUI' module. The INDUS code is open source and available on GitHub (https://github.com/patellab511/indus). , Matlab, GraphPad Prism, Excel, Image J

For manuscripts utilizing custom algorithms or software that are central to the research but not yet described in published literature, software must be made available to editors and reviewers. We strongly encourage code deposition in a community repository (e.g. GitHub). See the Nature Portfolio guidelines for submitting code & software for further information.

Data

Policy information about availability of data

All manuscripts must include a data availability statement. This statement should provide the following information, where applicable:

- Accession codes, unique identifiers, or web links for publicly available datasets
- A description of any restrictions on data availability
- For clinical datasets or third party data, please ensure that the statement adheres to our policy

The datasets generated in this study are available in the Figshare repository under https://doi.org/10.6084/m9.figshare.21509343.v3. UniProt Knowledgebase (UniProtKB) (Proteome ID UP000005640 [https://www.uniprot.org/proteomes/UP000005640], FUS, P35637 [https://www.uniprot.org/uniprotkb/P35637/entry], TAF15, Q92804 [https://www.uniprot.org/uniprotkb/Q92804/entry], EWS, Q01844 [https://www.uniprot.org/uniprotkb/Q01844/entry], hnRNPA1, P09651 [https://www.uniprot.org/uniprotkb/P09651/entry], G3BP1, Q13283 [https://www.uniprot.org/uniprotkb/Q13283/entry], FMRP1, Q06787 [https://www.uniprot.org/uniprotkb/Q06787/entry])

Human research participants

Policy information about studies involving human research participants and Sex and Gender in Research.

Reporting on sex and gender	not applicable
Population characteristics	not applicable
Recruitment	not applicable
Ethics oversight	not applicable

Note that full information on the approval of the study protocol must also be provided in the manuscript.

Field-specific reporting

Please select the one below that is the best fit for your research. If you are not sure, read the appropriate sections before making your selection.

- [x] Life sciences
- [ ] Behavioural & social sciences
- [ ] Ecological, evolutionary & environmental sciences

For a reference copy of the document with all sections, see nature.com/documents/nr-reporting-summary-flat.pdf

Life sciences study design

All studies must disclose on these points even when the disclosure is negative.

Sample size	Sample size was determined based on similar studies in this field.<br>For measuring viscosity and interfacial tension, between 7 and 14 imaging samples were analyzed. We determined this to be sufficient owing to low variability between samples and the data that fit well to the relations described in the Methods section.<br>Reference: Park, S., Barnes, R., Lin, Y. et al. Dehydration entropy drives liquid-liquid phase separation by molecular crowding. Commun Chem 3, 83 (2020). https://doi.org/10.1038/s42004-020-0328-8
Data exclusions	No data exclusion was conducted.
Replication	All the experiments were repeated at least twice or thrice, if not stated otherwise in the figure legends.<br>All attempts of replication were successful and gave similar results.
Randomization	Not relevant to this in vitro biophysical study. Samples for imaging were selected randomly.
Blinding	Blinding was not possible due to the inability to perform the in vitro characterization here presented if blinded.

Reporting for specific materials, systems and methods

We require information from authors about some types of materials, experimental systems and methods used in many studies. Here, indicate whether each material, system or method listed is relevant to your study. If you are not sure if a list item applies to your research, read the appropriate section before selecting a response.

Materials & experimental systems

n/a	Involved in the study
☒	☐ Antibodies
☒	☐ Eukaryotic cell lines
☒	☐ Palaeontology and archaeology
☒	☐ Animals and other organisms
☒	☐ Clinical data
☒	☐ Dual use research of concern

Methods

n/a	Involved in the study
☒	☐ ChIP-seq
☒	☐ Flow cytometry
☒	☐ MRI-based neuroimaging
